# Supplementary material for: The Landscape of Aberrant Alternative Splicing Events in Steatotic Liver Graft Post Transplantation via Transcriptome-Wide Analysis
Source: Int J Mol Sci. 2023 May 4;24(9):8216. doi: 10.3390/ijms24098216 (PMC10179559; doi:10.3390/ijms24098216)
Supplement: Supplementary file 1 [file ijms-24-08216-s001.zip › ijms-2263832-supplementary.pdf]

Supplementary figures

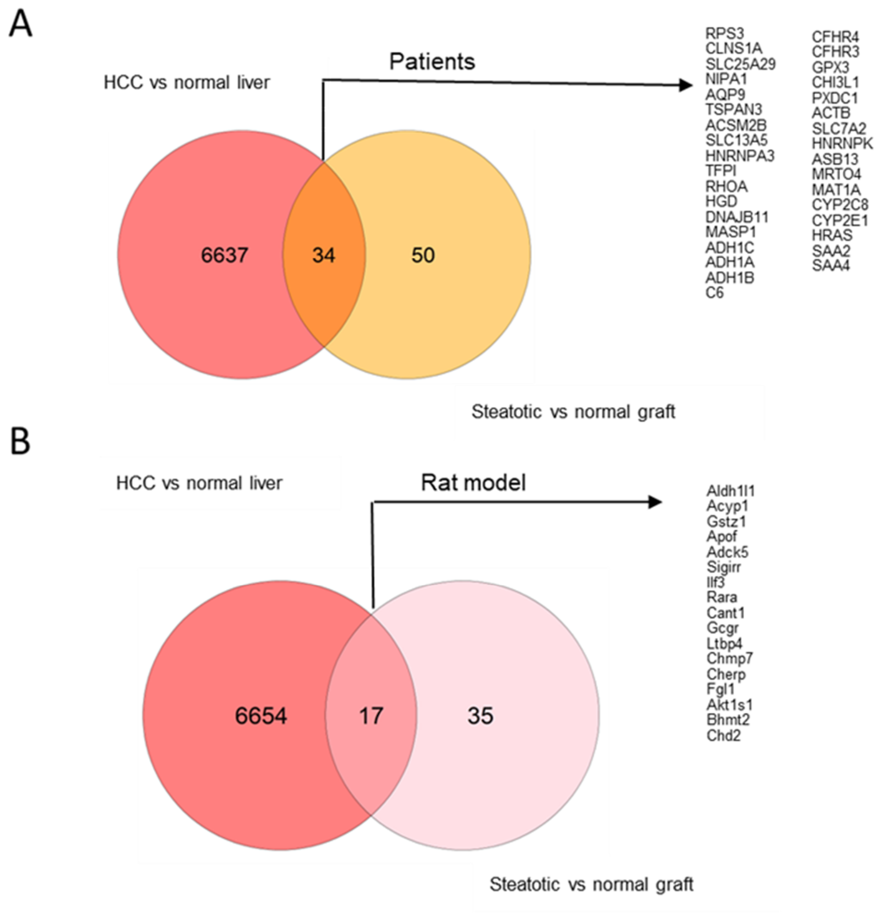

Figure S1. The overlapped genes involved differential ASEs of steatotic liver graft and HCC. **A** Thirty four genes in human were coincided in genes involved differential ASEs between HCC vs normal liver and steatotic vs normal liver graft. **B** Seventeen genes in rat model were found to be overlapped in genes involved differential ASEs between HCC vs normal liver and steatotic vs normal liver graft. ASEs, alternative splicing events.

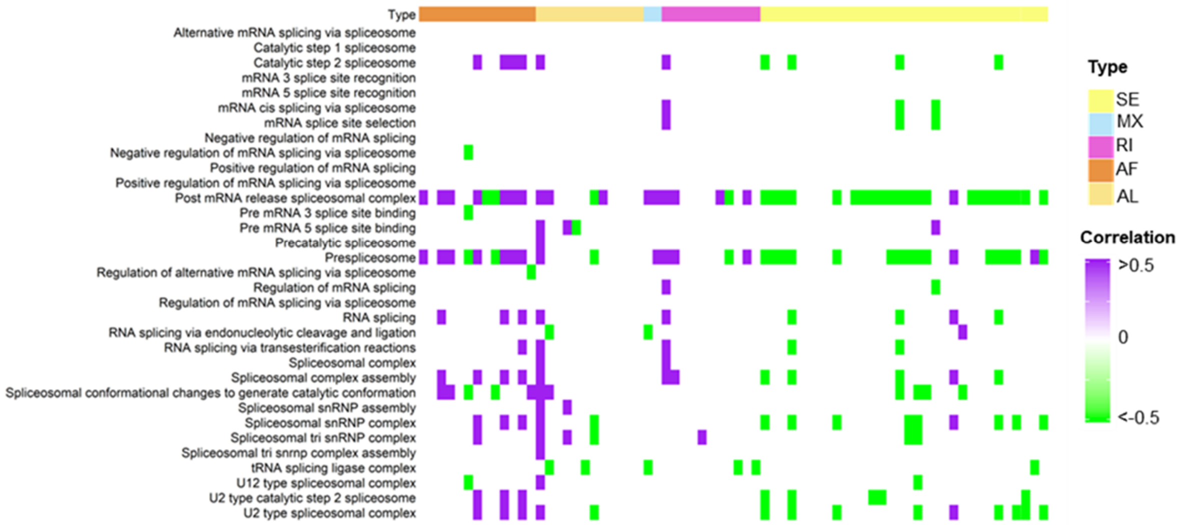

Figure S2. The differential ASEs were associated splicing factors. The association analysis of splicing factors and differential ASEs. ASEs, alternative splicing events.

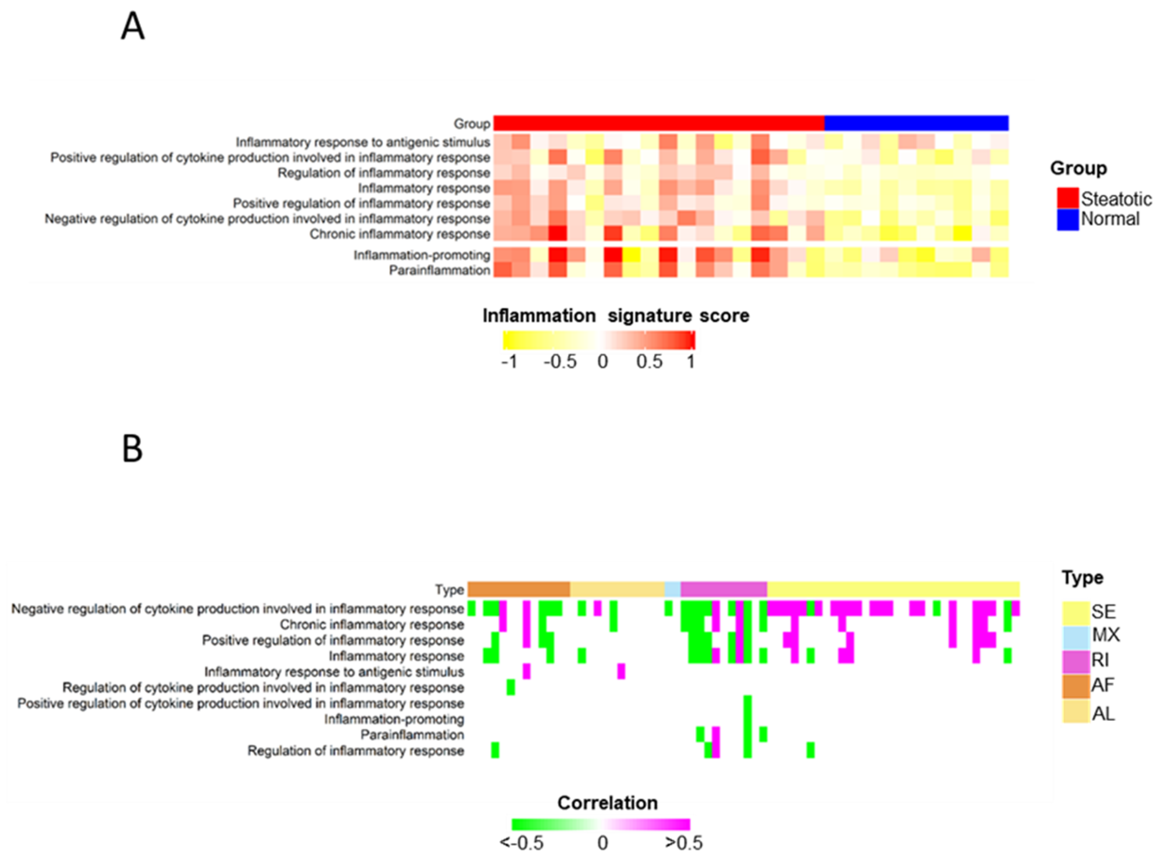

Figure S3. The severer inflammation in steatotic grafts were affected by differential ASEs. **A** The genes of inflammation siganature were enriched in human steatotic liver grafts. **B** The inflammation signature was affected differential ASEs. ASEs, alternative splicing events.

Supplementary table

Table S1. Characteristics of the patients post liver transplantation using normal or steatotic graft

	Normal graft (N=10)	Steatotic graft (N=18)
**Age (year)**	53.8	54.9
**Gender (Male)**	90%	94.4%
**Body weight (kg)**	68.8	69.4
**Alcoholic (Yes)**	0	0
**Hepatitis B positive**	90%	83.3%
**Hepatitis C positive**	10%	11.1%
**HCC**	100%	94.4%
**Fatty change**		
No (0%)	100%	0
Mild (<10%)	0	55.56%
Moderate (10-30%)	0	22.22%
Severe (>30%)	0	22.22%
**Cirrhosis**	90%	88.9%
**Living donor**	100%	83.3%
**Child-pugh score**	7	7.33
**Milan criteria (within criteria)**	70%	61.1%
**UCSF criteria (within criteria)**	70%	77.78%
**Graft weight (gm)**	600	1126
**Graft weight to Recipient ESLV (%)**	48.6	90.7
**Cold ischemia time (min)**	107	326
**SGOT**	514.9	830.6
**SGPT**	455.2	441.4
**Total bilirubin**	66.3	68.3

ESLV: Estimated Standard Liver Volume
